# *N*′-[(*E*)-2-Hy­droxy-5-iodo­benzyl­idene]furan-2-carbohydrazide monohydrate

**DOI:** 10.1107/S1600536811055826

**Published:** 2012-01-14

**Authors:** Rahman Bikas, Parisa Mahboubi Anarjan, Seik Weng Ng, Edward R. T. Tiekink

**Affiliations:** a Young Researchers Club, Tabriz Branch, Islamic Azad University, Tabriz, Iran; b Department of Chemistry, University of Zanjan, 45195-313 Zanjan, Iran; c Department of Chemistry, University of Malaya, 50603 Kuala Lumpur, Malaysia; d Chemistry Department, Faculty of Science, King Abdulaziz University, PO Box 80203 Jeddah, Saudi Arabia

## Abstract

The organic mol­ecule of the title monohydrate, C_12_H_9_IN_2_O_3_·H_2_O, features a disordered furyl ring with the major component [site occupancy = 0.575 (18)] having the carbonyl O and furyl O atoms *syn*, and the other conformation having these atoms *anti*. The mol­ecule is slightly twisted with the dihedral angle between the benzene and furyl rings being 10.3 (6)° (major component). An intra­molecular O—H⋯N(imine) hydrogen bond is formed. In the crystal, the water mol­ecule accepts a hydrogen bond from an amine H atom, and forms two O—H⋯O(carbon­yl) hydrogen bonds, thereby linking three different carbohydrazide mol­ecules. The result is a supra­molecular layer parallel to (001). The closest contacts between layers are of the type I⋯I, at a distance of 3.6986 (6) Å.

## Related literature

For historical background to aroylhydrazones, see: Craliz *et al.* (1955 ▶). For the structure of the isomorphous bromido deriv­ative, see: Tai *et al.* (2007 ▶). For the structures of related carbohydrazides, see: Abdel-Aziz *et al.* (2011 ▶); Bikas *et al.* (2012 ▶). For the synthesis of a precursor mol­ecule, see: Nielsen & Gothelf (2001 ▶).
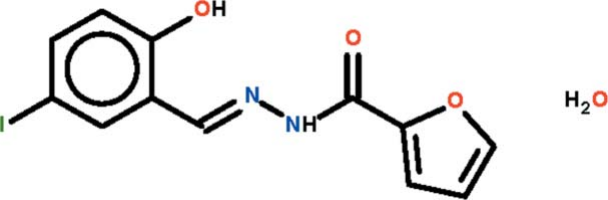


## Experimental

### 

#### Crystal data

C_12_H_9_IN_2_O_3_·H_2_O
*M* *_r_* = 374.13
Orthorhombic, 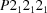

*a* = 4.8607 (2) Å
*b* = 12.5873 (4) Å
*c* = 21.1627 (9) Å
*V* = 1294.80 (9) Å^3^
*Z* = 4
Cu *K*α radiation
μ = 19.57 mm^−1^
*T* = 100 K
0.20 × 0.08 × 0.04 mm

#### Data collection

Agilent SuperNova Dual diffractometer with an Atlas detector
Absorption correction: multi-scan (*CrysAlis PRO*; Agilent, 2010 ▶) *T*_min_ = 0.111, *T*_max_ = 0.508
4758 measured reflections
2641 independent reflections
2575 reflections with *I* > 2σ(*I*)
*R*_int_ = 0.039

#### Refinement

*R*[*F* ^2^ > 2σ(*F* ^2^)] = 0.059
*wR*(*F* ^2^) = 0.158
*S* = 1.09
2641 reflections
186 parameters
34 restraints
H-atom parameters constrained
Δρ_max_ = 3.22 e Å^−3^
Δρ_min_ = −1.82 e Å^−3^
Absolute structure: Flack (1983 ▶), 1050 Friedel pairs
Flack parameter: −0.020 (12)

### 

Data collection: *CrysAlis PRO* (Agilent, 2010 ▶); cell refinement: *CrysAlis PRO*; data reduction: *CrysAlis PRO*; program(s) used to solve structure: *SHELXS97* (Sheldrick, 2008 ▶); program(s) used to refine structure: *SHELXL97* (Sheldrick, 2008 ▶); molecular graphics: *X-SEED* (Barbour, 2001 ▶) and *DIAMOND* (Brandenburg, 2006 ▶); software used to prepare material for publication: *publCIF* (Westrip, 2010 ▶).

## Supplementary Material

Crystal structure: contains datablock(s) global, I. DOI: 10.1107/S1600536811055826/hb6573sup1.cif

Structure factors: contains datablock(s) I. DOI: 10.1107/S1600536811055826/hb6573Isup2.hkl

Supplementary material file. DOI: 10.1107/S1600536811055826/hb6573Isup3.cml

Additional supplementary materials:  crystallographic information; 3D view; checkCIF report

## Figures and Tables

**Table 1:** Hydrogen-bond geometry (Å, °)

*D*—H⋯*A*	*D*—H	H⋯*A*	*D*⋯*A*	*D*—H⋯*A*
N2—H2⋯O1w	0.88	1.96	2.813 (7)	163
O1—H1⋯N1	0.84	2.20	2.744 (8)	122
O1w—H11⋯O2^i^	0.84	1.99	2.815 (8)	167
O1w—H12⋯O2^ii^	0.84	2.00	2.826 (8)	168

Symmetry codes: (i) 
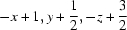
; (ii) 
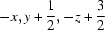
.

**Fig. 1.**
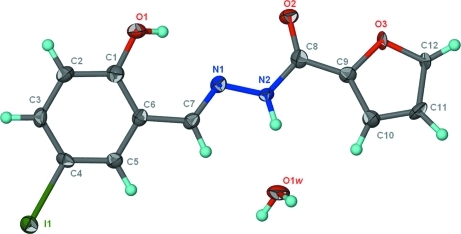
Molecular structure of (I) with displacement ellipsoids at the 70% probability level; hydrogen atoms are drawn as spheres of arbitrary radius. For reasons of clarity, only the major component of the furyl ring is shown.

**Fig. 2.**
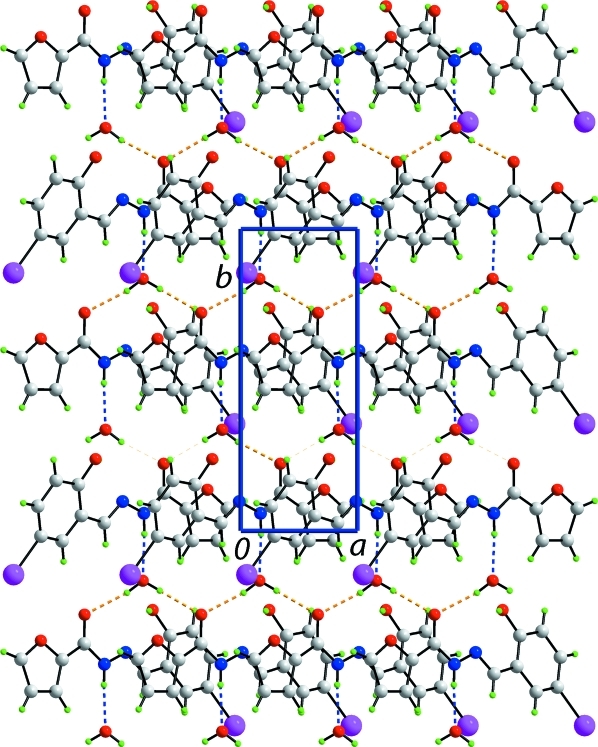
Supramolecular layer parallel to (001) in (I). The intermolecular O—H···O and N—H···O hydrogen bonds are shown as orange and blue dashed lines, respectively. For reasons of clarity, only the major component of the furyl ring is shown.

**Fig. 3.**
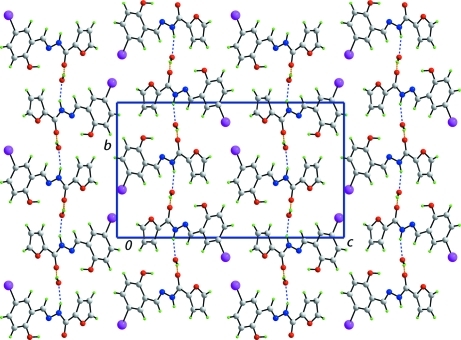
A view of the unit-cell contents of (I) in projection down the a axis. The intra- and inter-molecular O—H···O and the N—H···O hydrogen bonds are shown as orange and blue dashed lines, respectively. For reasons of clarity, only the major component of the furyl ring is shown.

## supplementary crystallographic information

### Comment

Hydrazone ligands (carbohydrazides), a class of Schiff base, derived from the condensation of acid hydrazides (R–CO–NH–NH_2_) with aromatic 2-hydroxy aldehydes or ketones (Craliz *et al.*, 1955) are potentially important tridentate *O*,*N*,*O*-donor ligands. Previous structural studies of carbohydrazide derivatives (Abdel-Aziz *et al.*, 2011; Bikas *et al.*, 2012) have been extended to include the title compound, (I), which is isomorphous with bromido derivative (Tai *et al.*, 2007).

In the molecule of (I), Fig. 1, the furyl ring was found to be disordered over two almost diagonally opposed orientations with the dihedral angle between the two components being 9.5 (10)°. In the major component (site occupancy = 0.575 (18)), the carbonyl-O and furyl-O atoms are *syn*. Overall, the molecule of (I) exhibits a small twist with the dihedral angle between the benzene and furyl rings being 10.3 (6)° for the major component; the comparable angle involving the minor component = 15.9 (8)°. The hydroxyl-H atom forms an intramolecular hydrogen bond to the imine-H atom, Table 1. The conformation about the C7═N1 imine bond [1.283 (10) Å] is *E*.

In the crystal packing, the amine-H atoms forms a hydrogen bond to the water-O, and the water-H atoms form hydrogen bonds to carbonyl-O atoms from two different molecules, Table 1. The result is a supramolecular layers parallel to (001) comprising alternating rows of carbohydrazide molecules and water molecules, Fig. 2. The layers stack along the *c* axis with the closest contacts between them being I···I interactions [3.6986 (6) Å for symmetry operation: -1/2 + *x*, 5/2 - *y*, 2 - *z*], Fig. 3.

### Experimental

2-Hydroxy-5-iodobenzaldehyde was synthesized according to the reported procedure by Nielsen & Gothelf (2001). For preparing the title compound a methanol (10 ml) solution of 2-hydroxy-5-iodobenzaldehyde (1.5 mmol) was added drop-wise to a methanol solution (10 ml) of 2-furanecarboxylic acid hydrazide (1.5 mmol), and the mixture was refluxed for 3 h. The solution was then evaporated on a steam bath to 5 cm^3^ and cooled to room temperature. The light-yellow precipitates of the title compound were separated and filtered off, washed with 3 ml of cooled methanol and then dried in air. Colourless crystals were obtained from its methanol:water (98:2 *v*/*v*) solution by slow solvent evaporation. Yield: 86%. IR (cm^-1^): 3447 (w, broad, —OH), 3262 (m, N—H); 1668 (*versus*, C═ O); 1609 (s, C═N(azomethine)); 952 (m, N—N); 1274, 1351 (*versus*, C—O enolate). ^1^H NMR (250.13 MHz; DMSO-d6): δ 12.16 (s, 1H, CO—NH—); 11.15 (s, 1H, —OH); 8.57 (s, 1H); 7.92 (s, 1H); 7.88 (s, 1H); 7.51 (d, 1H, J = 8.5 Hz); 7.30 (s, 1H); 7.74 (d, 1H, J = 8.75 Hz; 6.67 (s, 1H) p.p.m.. ^1^H NMR (250.13 MHz; DMSO-d6 + D2O): δ 8.85 (s, 1H); 7.83 (s, 1H); 7382 (s, 1H); 7.24 (s, 1H); 6.72 (d, 1H, J = 8.75 Hz); 6.64 (s, 1H) p.p.m.. ^13^C NMR (DMSO; 62.90 MHz): δ 81.72, 112.61, 115.82, 119.48, 122.35, 136.62, 139.76, 145.99, 146.52, 146.63, 154.56 and 157.35 p.p.m..

### Refinement

Carbon-bound H-atoms were placed in calculated positions [C—H 0.95 Å, N—H 0.88 Å, O—H 0.84%A; *U*_iso_(H) 1.2*U*_eq_(C,*N*,*O*)] and were included in the refinement in the riding model approximation.

The H-atoms of the water molecule were placed in chemically sensible positions on the basis of hydrogen bonds but were not refined; *U*_iso_(H) = 1.5*U*_eq_(O).

The furyl ring is disordered over two positions in a 0.575 (18): 0.425 (18) ratio. The C—O distances were restrained to 1.37±0.01 Å, the carbon–carbon single-bond distances to 1.42±0.01 Å and the carbon–carbon double-bond distances to 1.34±0.01 Å. The α-carbon atom is ordered. The *U*_iso_ of the atoms comprising the minor component were set to *U*_eq_ of those of the atoms of the major component which were refined anisotropically.

The final difference Fourier map had a peak at approximately 1 Å from I1 and a hole at approximately 1 Å from I1.

### Crystal data

**Table:**

C_12_H_9_IN_2_O_3_·H_2_O	*F*(000) = 728
*M**_r_* = 374.13	*D*_x_ = 1.919 Mg m^−^^3^
Orthorhombic, *P*2_1_2_1_2_1_	Cu *K*α radiation, λ = 1.54184 Å
Hall symbol: P 2ac 2ab	Cell parameters from 2924 reflections
*a* = 4.8607 (2) Å	θ = 4.1–76.3°
*b* = 12.5873 (4) Å	µ = 19.57 mm^−^^1^
*c* = 21.1627 (9) Å	*T* = 100 K
*V* = 1294.80 (9) Å^3^	Prism, colourless
*Z* = 4	0.20 × 0.08 × 0.04 mm

### Data collection

**Table:**

Agilent SuperNova Dual diffractometer with an Atlas detector	2641 independent reflections
Radiation source: SuperNova (Cu) X-ray Source	2575 reflections with *I* > 2σ(*I*)
Mirror	*R*_int_ = 0.039
Detector resolution: 10.4041 pixels mm^-1^	θ_max_ = 76.5°, θ_min_ = 4.1°
ω scan	*h* = −6→5
Absorption correction: multi-scan (CrysAlis PRO; Agilent, 2010)	*k* = −15→10
*T*_min_ = 0.111, *T*_max_ = 0.508	*l* = −18→26
4758 measured reflections	

### Refinement

**Table:**

Refinement on *F*^2^	Secondary atom site location: difference Fourier map
Least-squares matrix: full	Hydrogen site location: inferred from neighbouring sites
*R*[*F*^2^ > 2σ(*F*^2^)] = 0.059	H-atom parameters constrained
*wR*(*F*^2^) = 0.158	*w* = 1/[σ^2^(*F*_o_^2^) + (0.127*P*)^2^] where *P* = (*F*_o_^2^ + 2*F*_c_^2^)/3
*S* = 1.09	(Δ/σ)_max_ = 0.001
2641 reflections	Δρ_max_ = 3.22 e Å^−^^3^
186 parameters	Δρ_min_ = −1.82 e Å^−^^3^
34 restraints	Absolute structure: Flack (1983), 1050 Friedel pairs
Primary atom site location: structure-invariant direct methods	Flack parameter: −0.020 (12)

### Fractional atomic coordinates and isotropic or equivalent isotropic displacement parameters (Å^2^)

**Table:**

	*x*	*y*	*z*	*U*_iso_*/*U*_eq_	Occ. (<1)
I1	1.44774 (10)	1.14557 (3)	0.97807 (2)	0.0213 (2)	
O1	0.7447 (14)	0.7625 (4)	0.8846 (3)	0.0239 (12)	
H1	0.7161	0.7655	0.8455	0.036*	
O2	0.1609 (12)	0.7779 (4)	0.7326 (3)	0.0205 (10)	
O3	−0.214 (2)	0.8642 (7)	0.6509 (4)	0.016 (2)	0.575 (18)
O3'	−0.043 (3)	1.0345 (7)	0.6855 (7)	0.016*	0.425 (18)
O1W	0.3405 (13)	1.1654 (4)	0.7575 (3)	0.0265 (12)	
H11	0.4756	1.2055	0.7641	0.040*	
H12	0.1948	1.1982	0.7664	0.040*	
N1	0.5129 (13)	0.9031 (5)	0.8010 (3)	0.0170 (12)	
N2	0.3308 (13)	0.9420 (4)	0.7566 (3)	0.0158 (11)	
H2	0.3265	1.0105	0.7483	0.019*	
C1	0.8927 (16)	0.8472 (6)	0.9027 (3)	0.0196 (14)	
C2	1.0829 (17)	0.8331 (6)	0.9520 (4)	0.0222 (14)	
H2A	1.1050	0.7648	0.9704	0.027*	
C3	1.2403 (16)	0.9185 (6)	0.9745 (3)	0.0196 (14)	
H3	1.3645	0.9086	1.0087	0.024*	
C4	1.2132 (15)	1.0178 (6)	0.9464 (3)	0.0168 (13)	
C5	1.0320 (17)	1.0338 (5)	0.8971 (3)	0.0176 (13)	
H5	1.0188	1.1018	0.8779	0.021*	
C6	0.8665 (15)	0.9496 (6)	0.8753 (3)	0.0165 (13)	
C7	0.6706 (16)	0.9737 (6)	0.8255 (3)	0.0168 (13)	
H7	0.6588	1.0447	0.8105	0.020*	
C8	0.1574 (17)	0.8747 (6)	0.7254 (3)	0.0184 (13)	
C9	−0.0322 (15)	0.9270 (5)	0.6827 (3)	0.0166 (13)	
C10	−0.085 (3)	1.0323 (8)	0.6628 (8)	0.024 (3)	0.575 (18)
H10	0.0141	1.0934	0.6761	0.028*	0.575 (18)
C10'	−0.226 (5)	0.8865 (16)	0.6390 (12)	0.024*	0.425 (18)
H10'	−0.2631	0.8141	0.6297	0.028*	0.425 (18)
C11	−0.298 (3)	1.0318 (11)	0.6217 (7)	0.021 (3)	0.575 (18)
H11A	−0.3751	1.0920	0.6012	0.025*	0.575 (18)
C11'	−0.345 (4)	0.9727 (17)	0.6138 (9)	0.021*	0.425 (18)
H11'	−0.4821	0.9720	0.5819	0.025*	0.425 (18)
C12	−0.384 (3)	0.9257 (11)	0.6150 (5)	0.018 (3)	0.575 (18)
H12A	−0.5336	0.9012	0.5901	0.022*	0.575 (18)
C12'	−0.233 (4)	1.0650 (16)	0.6422 (9)	0.018*	0.425 (18)
H12'	−0.2832	1.1362	0.6325	0.022*	0.425 (18)

### Atomic displacement parameters (Å^2^)

**Table:**

	*U*^11^	*U*^22^	*U*^33^	*U*^12^	*U*^13^	*U*^23^
I1	0.0259 (3)	0.0187 (3)	0.0192 (3)	0.00161 (17)	−0.00272 (17)	−0.00437 (15)
O1	0.036 (3)	0.015 (2)	0.021 (3)	−0.005 (2)	−0.005 (2)	0.002 (2)
O2	0.022 (2)	0.013 (2)	0.026 (3)	0.002 (2)	−0.001 (2)	−0.0006 (19)
O3	0.023 (4)	0.018 (4)	0.006 (4)	0.004 (4)	−0.006 (3)	−0.004 (3)
O1W	0.027 (3)	0.013 (2)	0.039 (3)	−0.001 (2)	−0.002 (3)	−0.004 (2)
N1	0.023 (3)	0.017 (2)	0.012 (2)	0.002 (2)	0.001 (2)	−0.001 (2)
N2	0.022 (3)	0.010 (2)	0.015 (2)	0.001 (2)	−0.003 (2)	−0.001 (2)
C1	0.024 (4)	0.019 (3)	0.016 (3)	−0.002 (3)	0.001 (3)	0.000 (3)
C2	0.032 (4)	0.016 (3)	0.019 (3)	0.000 (3)	−0.004 (3)	0.003 (3)
C3	0.023 (3)	0.024 (4)	0.012 (3)	0.000 (3)	−0.004 (3)	0.003 (3)
C4	0.021 (3)	0.014 (3)	0.015 (3)	−0.002 (3)	0.001 (3)	−0.006 (2)
C5	0.026 (3)	0.014 (3)	0.012 (3)	0.001 (3)	0.000 (3)	−0.002 (2)
C6	0.016 (3)	0.017 (3)	0.016 (3)	0.003 (3)	0.000 (3)	0.001 (2)
C7	0.021 (3)	0.016 (3)	0.013 (3)	0.002 (3)	0.001 (3)	0.001 (2)
C8	0.020 (3)	0.020 (3)	0.015 (3)	0.003 (3)	0.001 (3)	−0.002 (3)
C9	0.021 (3)	0.014 (3)	0.015 (3)	0.003 (3)	0.001 (3)	−0.005 (2)
C10	0.031 (7)	0.019 (5)	0.020 (6)	0.000 (5)	−0.007 (6)	−0.003 (5)
C11	0.025 (6)	0.018 (6)	0.021 (6)	0.005 (5)	−0.002 (5)	0.005 (5)
C12	0.029 (6)	0.019 (6)	0.007 (4)	−0.003 (5)	−0.007 (4)	0.000 (4)

### Geometric parameters (Å, °)

**Table:**

I1—C4	2.082 (7)	C3—H3	0.9500
O1—C1	1.342 (9)	C4—C5	1.379 (10)
O1—H1	0.8400	C5—C6	1.409 (11)
O2—C8	1.228 (9)	C5—H5	0.9500
O3—C9	1.363 (8)	C6—C7	1.451 (10)
O3—C12	1.363 (9)	C7—H7	0.9500
O3'—C9	1.355 (8)	C8—C9	1.449 (10)
O3'—C12'	1.358 (9)	C9—C10	1.413 (9)
O1W—H11	0.8402	C9—C10'	1.413 (10)
O1W—H12	0.8409	C10—C11	1.353 (9)
N1—C7	1.283 (10)	C10—H10	0.9500
N1—N2	1.381 (8)	C10'—C11'	1.341 (10)
N2—C8	1.365 (10)	C10'—H10'	0.9500
N2—H2	0.8800	C11—C12	1.407 (9)
C1—C2	1.406 (10)	C11—H11A	0.9500
C1—C6	1.418 (10)	C11'—C12'	1.415 (10)
C2—C3	1.402 (10)	C11'—H11'	0.9500
C2—H2A	0.9500	C12—H12A	0.9500
C3—C4	1.391 (9)	C12'—H12'	0.9500
			
C1—O1—H1	109.5	C6—C7—H7	118.6
C9—O3—C12	109.8 (8)	O2—C8—N2	123.1 (7)
C9—O3'—C12'	106.2 (12)	O2—C8—C9	122.5 (7)
H11—O1W—H12	109.0	N2—C8—C9	114.4 (6)
C7—N1—N2	114.4 (6)	O3—C9—C10	106.3 (8)
C8—N2—N1	120.4 (6)	O3'—C9—C10'	111.3 (11)
C8—N2—H2	119.8	O3'—C9—C8	116.8 (8)
N1—N2—H2	119.8	O3—C9—C8	117.1 (6)
O1—C1—C2	117.6 (6)	C10—C9—C8	136.6 (8)
O1—C1—C6	123.9 (7)	C10'—C9—C8	131.8 (10)
C2—C1—C6	118.5 (7)	C11—C10—C9	109.0 (9)
C3—C2—C1	120.9 (6)	C11—C10—H10	125.5
C3—C2—H2A	119.5	C9—C10—H10	125.5
C1—C2—H2A	119.5	C11'—C10'—C9	104.7 (14)
C4—C3—C2	119.5 (7)	C11'—C10'—H10'	127.6
C4—C3—H3	120.3	C9—C10'—H10'	127.6
C2—C3—H3	120.3	C10—C11—C12	107.2 (10)
C5—C4—C3	121.0 (6)	C10—C11—H11A	126.4
C5—C4—I1	118.7 (5)	C12—C11—H11A	126.4
C3—C4—I1	120.3 (5)	C10'—C11'—C12'	109.3 (16)
C4—C5—C6	120.2 (6)	C10'—C11'—H11'	125.3
C4—C5—H5	119.9	C12'—C11'—H11'	125.3
C6—C5—H5	119.9	O3—C12—C11	107.6 (9)
C5—C6—C1	119.9 (7)	O3—C12—H12A	126.2
C5—C6—C7	117.1 (6)	C11—C12—H12A	126.2
C1—C6—C7	123.0 (7)	O3'—C12'—C11'	108.3 (15)
N1—C7—C6	122.7 (6)	O3'—C12'—H12'	125.8
N1—C7—H7	118.6	C11'—C12'—H12'	125.8
			
C7—N1—N2—C8	177.9 (6)	C12—O3—C9—C10'	−21 (6)
O1—C1—C2—C3	−178.5 (7)	C12—O3—C9—C8	177.4 (9)
C6—C1—C2—C3	1.0 (11)	O2—C8—C9—O3'	170.8 (9)
C1—C2—C3—C4	−1.8 (12)	N2—C8—C9—O3'	−9.3 (11)
C2—C3—C4—C5	0.6 (11)	O2—C8—C9—O3	1.1 (12)
C2—C3—C4—I1	−179.3 (6)	N2—C8—C9—O3	−179.0 (8)
C3—C4—C5—C6	1.3 (11)	O2—C8—C9—C10	−178.1 (12)
I1—C4—C5—C6	−178.8 (5)	N2—C8—C9—C10	1.8 (16)
C4—C5—C6—C1	−2.0 (11)	O2—C8—C9—C10'	−5(2)
C4—C5—C6—C7	176.9 (7)	N2—C8—C9—C10'	174.5 (19)
O1—C1—C6—C5	−179.6 (7)	O3'—C9—C10—C11	−151 (3)
C2—C1—C6—C5	0.8 (11)	O3—C9—C10—C11	1.9 (15)
O1—C1—C6—C7	1.6 (12)	C10'—C9—C10—C11	6.6 (18)
C2—C1—C6—C7	−178.0 (7)	C8—C9—C10—C11	−178.8 (10)
N2—N1—C7—C6	176.9 (6)	O3'—C9—C10'—C11'	3(3)
C5—C6—C7—N1	178.6 (7)	O3—C9—C10'—C11'	157 (8)
C1—C6—C7—N1	−2.6 (11)	C10—C9—C10'—C11'	−6(2)
N1—N2—C8—O2	−3.3 (11)	C8—C9—C10'—C11'	179.0 (13)
N1—N2—C8—C9	176.8 (6)	C9—C10—C11—C12	0.0 (17)
C12'—O3'—C9—O3	−10.9 (17)	C9—C10'—C11'—C12'	−1(3)
C12'—O3'—C9—C10	21 (2)	C9—O3—C12—C11	3.2 (14)
C12'—O3'—C9—C10'	−3(2)	C10—C11—C12—O3	−1.9 (16)
C12'—O3'—C9—C8	−179.6 (11)	C9—O3'—C12'—C11'	1.6 (19)
C12—O3—C9—O3'	8.7 (15)	C10'—C11'—C12'—O3'	0(3)
C12—O3—C9—C10	−3.1 (13)		

### Hydrogen-bond geometry (Å, °)

**Table:**

*D*—H···*A*	*D*—H	H···*A*	*D*···*A*	*D*—H···*A*
N2—H2···O1w	0.88	1.96	2.813 (7)	163
O1—H1···N1	0.84	2.20	2.744 (8)	122
O1w—H11···O2^i^	0.84	1.99	2.815 (8)	167
O1w—H12···O2^ii^	0.84	2.00	2.826 (8)	168

Symmetry codes: (i) −*x*+1, *y*+1/2, −*z*+3/2; (ii) −*x*, *y*+1/2, −*z*+3/2.
